# Phase-Field Modeling of Chemoelastic Binodal/Spinodal Relations and Solute Segregation to Defects in Binary Alloys

**DOI:** 10.3390/ma14071787

**Published:** 2021-04-05

**Authors:** Jaber Rezaei Mianroodi, Pratheek Shanthraj, Bob Svendsen, Dierk Raabe

**Affiliations:** 1 Microstructure Physics and Alloy Design, Max-Planck-Institut für Eisenforschung GmbH, 40237 Düsseldorf, Germany or bob.svendsen@rwth-aachen.de (B.S.); d.raabe@mpie.de (D.R.); 2 The Department of Materials, The University of Manchester, Manchester M13 9PL, UK; pratheek.shanthraj@manchester.ac.uk; 3 Material Mechanics, RWTH Aachen University, 52062 Aachen, Germany

**Keywords:** phase-field chemomechanics, solute segregation, spinodal decomposition, dislocation-solute interaction, low angle grain boundary

## Abstract

Microscopic phase-field chemomechanics (MPFCM) is employed in the current work to model solute segregation, dislocation-solute interaction, spinodal decomposition, and precipitate formation, at straight dislocations and configurations of these in a model binary solid alloy. In particular, (i) a single static edge dipole, (ii) arrays of static dipoles forming low-angle tilt (edge) and twist (screw) grain boundaries, as well as at (iii) a moving (gliding) edge dipole, are considered. In the first part of the work, MPFCM is formulated for such an alloy. Central here is the MPFCM model for the alloy free energy, which includes chemical, dislocation, and lattice (elastic), contributions. The solute concentration-dependence of the latter due to solute lattice misfit results in a strong elastic influence on the binodal (i.e., coexistence) and spinodal behavior of the alloy. In addition, MPFCM-based modeling of energy storage couples the thermodynamic forces driving (Cottrell and Suzuki) solute segregation, precipitate formation and dislocation glide. As implied by the simulation results for edge dislocation dipoles and their configurations, there is a competition between (i) Cottrell segregation to dislocations resulting in a uniform solute distribution along the line, and (ii) destabilization of this distribution due to low-dimensional spinodal decomposition when the segregated solute content at the line exceeds the spinodal value locally, i.e., at and along the dislocation line. Due to the completely different stress field of the screw dislocation configuration in the twist boundary, the segregated solute distribution is immediately unstable and decomposes into precipitates from the start.

## 1. Introduction

The dependence of the material properties on chemical composition, temperature and pressure (stress) is central to the phase relations, thermodynamics and behavior of many materials. In the case of engineering alloys, for example, the dependence of elastic or magnetic material properties on chemical composition can have a significant influence on alloy thermodynamics, phase relations, and mechanical behavior due for example to defects such as dislocations.

A classical example of the latter, going back to the seminal work of Cottrell (e.g., Cottrell and Bilby [1]; see also Hirth and Lothe [2]), is the composition dependence of lattice distortion due to solute misfit, resulting in a contribution of the stress field to the solute chemical potential, and so to the driving force for solute diffusion, for example to lattice defects such as dislocations (e.g., Kuzmina et al. [3], Kwiatkowski da Silva et al. [4], Kwiatkowski da Silva et al. [5], Zhou et al. [6]). More recent work has focused on further aspects and details of this type of chemoelastic coupling. In Ma et al. [7], solute segregation and “wetting transition” at stationary and gliding dislocations has been investigated with the help of linear elastic phase-field microelasticity (PFM: e.g., Wang et al. [8], Wang and Li [9]) and semi-atomistic considerations. They conclude that short-range chemical interaction among solute atoms lies behind a “wetting transition” at the dislocation core, depending in particular on temperature and solute concentration.

Besides to the driving force for solute diffusion and segregation to defects, the composition dependence of lattice distortion due to solute misfit also leads to elastic effects on the alloy binodal and spinodal. In particular with respect to spinodal decomposition, this has been shown in the work of Cahn [10,11], and broadly generalized by Khachaturyan [12]. This includes both the energetics and kinetics of spinodal decomposition in defect-free systems, as dicussed for example by Fultz [13] (§12.5). A recent example of this is the work of Barkar et al. [14], who combined the linear chemoelastic model of Cahn [10] with a dependence of the gradient chemical energy on solute concentration due to magnetic transitions to model defect-free spinodal decomposition and precipitate formation in Fe-Cr. Further elastic effects on phase equilibria besides solute misfit have been investigated recently by Korbmacher et al. [15] for the defect-free binary system Ni-H. As their results demonstrate, in addition to solute misfit, both geometric and physical (i.e., anharmonic) elastic non-linearity have a significant influence for example on coexistence.

Purely atomistic approaches to the modeling of precipitation and second-phase formation include for example variance-control semi-grand canonical Monte Carlo molecular dynamics Sadigh et al. [16]. This approach has recently been employed by Turlo and Rupert [17,18] to simulate structural and chemical reordering at (bulk) dislocations in a number of vacancy-free binary fcc alloys, resulting in the formation of so-called linear complexions. By varying temperature and composition, they determined linear complexion diagrams analogous to bulk phase diagrams. In this fashion, they predicted a number of complexion types in different binary (and ternary) alloy systems. Formation of such linear complexions is expected to have a strong effect on material properties.

In the current work, microscopic phase-field chemomechanics (MPFCM) is employed to model microscopic dislocation-solute interaction in a generic binary alloy. This represents an application of the phase-field-based modeling methodology developed in Svendsen et al. [19] to the modeling of microscopic dislocation-solute interaction. In particular, this involves phase-field modeling of microscopic dislocations based on finite-deformation generalization of PFM and phase-field dislocation dynamics (e.g., Hunter et al. [20,21], Xu et al. [22]). Combination of this with alloy chemical thermodynamics yields MPFCM. Such models have been applied to for example to investigate microscopic dislocation-solute interaction and other processes in many other alloys (e.g., in Ni-based superalloys: Mianroodi et al. [23], Wu et al. [24]).

The work is organized as follows. In Section 2, the form of the MPFCM model for the binary solid alloy is briefly summarized. In particular, this is based on balance and constitutive relations, in particular for the free energy. The free energy model is based on elastic, dislocation, and chemical contributions. For simplicity, the chemical part is based on pairwise interaction and regular solution modeling. Simplification of this model to cubic alloys for use in simulations is discussed in Section 3. In particular, in this context, the regular solution model reduces to that of Cahn and Hilliard [25]. After discussing simulation details in Section 4, results are presented and discussed in detail in Section 5. These include (i) linear elastic effects on the alloy binodal and spinodal, as well as solute segregation to, and interaction with, (ii) stationary dipole edge dislocations, (iii) dislocation dipole arrays in low-angle tilt and twist boundaries, and (iv) gliding dislocation dipoles. The work ends with a summary in Section 6.

In this work, (three-dimensional) Euclidean vectors are represented by lower-case bold italic characters a,b,…. In particular, let $i_{1}$, $i_{2}$, and $i_{3}$ represent the Cartesian basis vectors. Second-order tensors are represented by upper-case bold italic characters A,B,…. Let I be the second-order identity. Third- and fourth-order Euclidean tensors A,B,… are denoted by upper-case slanted sans-serif characters. The context will make clear which order pertains. The scalar product A·B of two arbitrary-order tensors A and B is defined by $A\cdot B:=A_{ijk\ldots }B_{ijk\ldots }$ (contraction; sum on repeated indices). In particular, then, $a\cdot b=a_{i}\;b_{i}$ represents the scalar product of two vectors. Given this, $A^{T}b\cdot c:=b\cdot Ac$ defines the transpose $A^{T}$ of A, $\mathrm{sym}A:=\frac{1}{2}\left(A+A^{T}\right)$ its symmetric part. Let v and T be differentiable tensor fields. The curl of these can be defined by a·curlv:=div(v×a) and ${\left(\mathrm{curl}T\right)}^{T}a:=\mathrm{curl}\left(T^{T}a\right)$ with respect to any constant vector a. Additional notation and relations will be introduced as needed.

## 2. Basic Model Formulation

### 2.1. Balance and Basic Constitutive Relations

Consider a binary solid solution/alloy with solvent A and solute B. Basic unknowns of the current chemomechanical model for this alloy include the solute chemical concentration field $c=c_{B}$, the alloy deformation field χ, and the scalar phase fields $\phi =(\phi_{1},\ldots ,\phi_{g})$ modeling transitions between unslipped and slipped lattice states due to the presence and motion of dislocations. Restricting attention to isothermal (i.e., constant absolute temperature θ) and quasi-static conditions, and neglecting all supplies, the balance relations

(1) $$\dot{c}=-\mathrm{div}j\;,\;\;0=\mathrm{div}P\;,\;\;FP^{T}=PF^{T}\;,\;\;\dot{\varepsilon }=\mathrm{div}\;\left(P^{T}\dot{\chi }\right)\;,\;\;\dot{\eta }=\theta^{-1}\delta +\mathrm{div}\;\left(\theta^{-1}\mu j\right)\;,$$

hold for solute mass, alloy linear momentum, alloy angular momentum, alloy (internal) energy, and alloy entropy, respectively (e.g., [26] (Chapters 2–3); generalization to finite-deformation in Svendsen et al. [19]). In these relations, j is the solute concentration flux density (units m s${}^{-1}$), F:=∇χ is the deformation gradient (dimensionless), and P is the first Piola-Kirchhoff (PK) stress (units N m${}^{-2}$). Further, ε (units J m${}^{-3}$), η (units J m${}^{-3}$ K${}^{-1}$), and δ (units J m${}^{-3}$ s${}^{-1}$) represent the alloy’s internal energy, entropy, and dissipation-rate densities, respectively. Lastly, $\mu :=\mu_{B}-\mu_{A}$ is the solute (relative molar) chemical or diffusion potential (units J mol${}^{-1}$) of the solute.

In addition to the balance relations (1), the current model for binary alloy chemomechanics is based on the general constitutive form

(2) $$\psi \left(F,c,\phi ,\nabla c,\nabla \phi \right)\;,\;f:=\upsilon_{m}\psi \;,$$

for the free energy density ψ:=ε−θη. In this case, the molar form *f* of ψ is also determined, with $\upsilon_{m}$ the (here assumed constant) alloy molar volume (units m${}^{3}$ mol${}^{-1}$). Since attention is focused in this work on purely bulk behavior, the generalized no-flux boundary conditions

(3) $$\dot{c}\;\left(\partial_{\nabla c}\psi \right)\cdot n=0\;,\;\sum_{a}{\dot{\phi }}_{a}\;\left(\partial_{\nabla \phi_{a}}\psi \right)\cdot n=0\;,$$

hold on the boundary of any region with unit normal n. In addition, the dependent constitutive relations

(4) $$\mu =\delta_{c}f\;,\;P=\partial_{F}\psi ,\;j=-m_{c}\nabla \mu \;,\;{\dot{\phi }}_{a}=-m_{a}\;\delta_{\phi_{a}}\psi \;,$$

apply, with $\delta_{x}g:=\partial_{x}g-\mathrm{div}\partial_{\nabla x}g$ the variational derivative of *g*. Given non-negative dislocation $m_{a}$ (units J${}^{-1}$ m${}^{3}$ s${}^{-1}$) and solute $m_{c}$ (units J${}^{-1}$ mol m${}^{2}$ s${}^{-1}$) mobilities, (3) and (4) are sufficient for non-negative δ.

Analogous to the purely chemical case (e.g., Cahn and Hilliard [25]), the chemomechanical free energy density

(5) $$\psi \left(F,c,\phi ,\nabla c,\nabla \phi \right)=\psi_{\mathrm{ho}}\left(F,c,\phi \right)+\psi_{\mathrm{gr}}\left(\phi ,\nabla c,\nabla \phi \right)$$

in (2) is modeled here as the sum of “homogeneous” $\psi_{\mathrm{ho}}$ and “gradient” $\psi_{\mathrm{gr}}$ contributions. The former

(6) $$\psi_{\mathrm{ho}}\left(F,c,\phi \right)=\psi_{\mathrm{el}}\left(F,c,\phi \right)+\psi_{\mathrm{hd}}\left(c,\phi \right)+\psi_{\mathrm{hc}}\left(c\right)$$

is determined in general by elastic (lattice) $\psi_{\mathrm{el}}$, dislocation $\psi_{\mathrm{hd}}$, and chemical $\psi_{\mathrm{hc}}$, parts, respectively. The latter

(7) $$\psi_{\mathrm{gr}}\left(\phi ,\nabla c,\nabla \phi \right)=\psi_{\mathrm{gd}}\left(\phi ,\nabla \phi \right)+\psi_{\mathrm{gc}}\left(\nabla c\right)$$

consists in general of dislocation $\psi_{\mathrm{gd}}$ and chemical $\psi_{\mathrm{gc}}$ parts. The material properties determining $\psi_{\mathrm{gd}}$ are also generally dependent on *c*, but this is neglected here for simplicity. The form of $\psi_{\mathrm{gc}}$ is based for simplicity here on pairwise interaction and regular solution theory, in which case $\psi_{\mathrm{gc}}$ is independent of *c* (e.g., Cahn and Hilliard [25], Lass et al. [27]). All of these contributions to ψ are discussed in more detail in what follows.

### 2.2. Local Kinematics and Elastic Energy

Let

(8) $$F_{L}:=FF_{R}^{-1}\;,\;C_{L}:=F_{L}^{T}F_{L}\;,\;E_{L}:=\frac{1}{2}\left(C_{L}-I\right)\;,$$

be the lattice (elastic) local deformation, right Cauchy-Green deformation, and Green strain, respectively. The residual (i.e., zero-stress) local deformation

(9) $$F_{R}=F_{S}F_{D}\;,\;\begin{array}{ccc} F_{S}\left(c\right)\; & = & \exp\left[\left(c-c_{0}\right)\;N_{S}\right]\;F_{S}\left(c_{0}\right)\;,\\ F_{D}\left(\phi \right)\; & = & \exp\left[\sum_{a=1}^{g}\left(\phi_{a}-\phi_{a0}\right)\;N_{Da}\right]\;F_{D}\left(\phi_{0}\right)\;, \end{array}$$

in the current model is determined by contributions from solute misfit $F_{S}$ and dislocation motion $F_{D}$ on *g* systems a=1,…,g. Here, $N_{S}$ represents the (infinitesimal, linear) distortion per unit solute concentration due to solute misfit. Restricting attention to dislocation glide,

(10) $$N_{Da}=\gamma_{a}⊗n_{a}=\gamma_{a}\;s_{a}⊗n_{a}\;,$$

holds. Here, $\gamma_{a}:=b_{a}/d_{a}$, $\gamma_{a}:=b_{a}/d_{a}$, $b_{a}:=\left|b_{a}\right|$, $s_{a}:=b_{a}/b_{a}$, $b_{a}$ is the Burgers vector, $d_{a}$ the lattice slip plane spacing, and $n_{a}$ the slip plane normal. In the current work, attention is restricted to the special case that $s_{a}\cdot n_{b}=0$ for a≠b, resulting in the simplified form

(11) $$F_{D}\left(\phi \right)=\left[I+\sum_{a=1}^{g}\left(\phi_{a}-\phi_{a0}\right)\;\gamma_{a}⊗n_{a}\right]F_{D}\left(\phi_{0}\right)$$

of the third relation in (9) based on (10).

Assuming “small” lattice strain $|E_{L}|\ll 1$ (i.e., outside the dislocation core), the harmonic form

(12) $$\psi_{\mathrm{el}}\left(F,c,\phi \right)=\frac{1}{2}\;E_{L}\left(F,c,\phi \right)\cdot C_{\mathrm{el}}\;E_{L}\left(F,c,\phi \right)$$

for $\psi_{\mathrm{el}}$ applies, with $C_{\mathrm{el}}$ the elastic stiffness, assumed independent of *c* for simplicity. From (9) and (12) follow the forms

(13) $$\begin{array}{ccc} \partial_{c}\psi_{\mathrm{el}}\; & = & -N_{S}\cdot M=-F_{L}N_{S}F_{L}^{-1}\cdot K\;,\\ \partial_{\phi_{a}}\psi_{\mathrm{el}}\; & = & -\gamma_{a}\cdot Mn_{a}\;,\\ \partial_{c}^{2}\psi_{\mathrm{el}}\; & = & I\cdot C_{\mathrm{el}}\left[N_{S}\right]N_{S}+2E_{L}\cdot \left(2C_{\mathrm{el}}\left[N_{S}\right]N_{S}+C_{\mathrm{el}}\left[N_{S}N_{S}\right]\right)\\ \; & + & 4E_{L}\cdot \left(C_{\mathrm{el}}\left[E_{L}N_{S}\right]N_{S}+C_{\mathrm{el}}\left[E_{L}N_{S}N_{S}\right]\right)\;, \end{array}$$

for the concentration and phase-field derivatives of $\psi_{\mathrm{el}}$, where

(14) $$K:=PF^{T}=\left(\partial_{F}\psi_{\mathrm{el}}\right)F^{T}=F_{L}S_{L}F_{L}^{T}=F_{L}^{-T}MF_{L}^{T}\;,\;\;S_{L}:=\partial_{E_{L}}\psi_{\mathrm{el}}\;,\;\;M:=C_{L}S_{L}\;,$$

represent the Kirchhoff, “lattice” second PK, and Mandel, stresses, respectively.

For later purposes, it will be useful to compare this non-linear elastic model with its linear counterpart. As usual, this is based in particular on the displacement gradient H:=∇u=F−I and linear lattice distortion $H_{\mathrm{Ll}}:=H-H_{\mathrm{Rl}}$, with $H_{\mathrm{Rl}}=H_{\mathrm{Sl}}+H_{\mathrm{Dl}}=c\;N_{S}+\sum_{a=1}^{g}\phi_{a}\;\gamma_{a}⊗n_{a}$ corresponding to (9) with (10) in the non-linear case. Given these, the linear elastic energy density takes the form

(15) $$\psi_{\mathrm{le}}\left(E,c,\phi \right)=\frac{1}{2}\;E_{\mathrm{Ll}}\left(E,c,\phi \right)\cdot C_{\mathrm{el}}\;E_{\mathrm{Ll}}\left(E,c,\phi \right)\;,$$

with $E_{\mathrm{Ll}}:=\mathrm{sym}H_{\mathrm{Ll}}=E-E_{\mathrm{Rl}}$, and E:=symH the linear strain. From (15) follow

(16) $$\partial_{c}\psi_{\mathrm{le}}=-N_{S}\cdot T\;,\;\;\partial_{\phi_{a}}\psi_{\mathrm{le}}=-\gamma_{a}\cdot Tn_{a}\;,\;\;\partial_{c}^{2}\psi_{\mathrm{le}}=I\cdot C_{\mathrm{el}}\left[N_{S}\right]N_{S}\;,\;\;T=\partial_{E_{\mathrm{Ll}}}\psi_{\mathrm{le}}\;,$$

corresponding to (13) and (14) in the non-linear case, where T is the linear elastic stress. In contrast to the third relation in (13) for $\partial_{c}^{2}\psi_{\mathrm{el}}$ in the non-linear model, note that the third relation in (16) for $\partial_{c}^{2}\psi_{\mathrm{le}}$ in the linear case is independent of lattice strain. As discussed in more detail below, this has consequences for the system binodal and spinodal in the current chemomechanical context.

### 2.3. Dislocation Energy

In general, the homogeneous dislocation energy $\psi_{\mathrm{hd}}$ is related to energy barriers resulting in lattice resistance to dislocation transitions and motion. Examples of this include the stacking fault energy

(17) $$\psi_{\mathrm{hd}}\left(c,\phi \right)=\gamma_{\mathrm{sf}}\left(c,\phi \right)/d_{111}$$

with respect to {111} glide planes (of spacing $d_{111}=a_{0}/\sqrt{3}$) in the fcc case, or the energy related to (screw) core spreading in the bcc case.

Restricting attention to planar dislocation cores for simplicity, the dislocation core energy $\psi_{\mathrm{gd}}$ is modeled here by the simple quadratic from $\psi_{\mathrm{gd}}\left(\phi ,\nabla \phi \right)=\varphi_{\mathrm{gd}}\;{\left|G_{D}\left(\phi ,\nabla \phi \right)\right|}^{2}$ in terms of the gradient energy coefficient $\varphi_{\mathrm{gd}}$ (units J m${}^{-1}$) and dislocation tensor $G_{D}:=\mathrm{curl}F_{D}$. As already mentioned above, $\varphi_{\mathrm{gd}}$ will also depends on *c* in general, but this is neglected here for simplicity, and $\partial_{c}\psi_{\mathrm{gd}}=0$. Given further (11), $\partial_{\phi_{a}}\psi_{\mathrm{gd}}=2\;\varphi_{\mathrm{gd}}\;G_{D}^{T}\gamma_{a}\cdot G_{D0}^{T}n_{a}$ and $\partial_{\nabla \phi_{a}}\psi_{\mathrm{gd}}=2\;\varphi_{\mathrm{gd}}\;\left(F_{D0}^{T}n_{a}\right)\times \left(G_{D}^{T}\gamma_{a}\right)$ are linear in $G_{D}=G_{D0}+\sum_{a=1}^{g}\left(\phi_{a}-\phi_{a0}\right)\;\gamma_{a}⊗G_{D0}^{T}n_{a}+\sum_{a=1}^{g}\gamma_{a}⊗\nabla \left(\phi_{a}-\phi_{a0}\right)\times F_{D0}^{T}\;n_{a}$. Adopting the initial conditions $F_{D0}=I$, $\nabla \phi_{0}=0$, and $G_{D0}=0$, the linearized form $G_{D}\left(\nabla \phi \right)=\sum_{a=1}^{g}\gamma_{a}⊗\nabla \phi_{a}\times \;n_{a}$ of $G_{D}$ holds. On this basis, the simplified model relations

(18) $$\begin{array}{ccc} \psi_{\mathrm{gd}}\left(\nabla \phi \right)\; & = & \varphi_{\mathrm{gd}}\;\sum_{a,b=1}^{g}\nabla \phi_{a}\cdot \left(\gamma_{a}\cdot \gamma_{b}\right)\left[\left(n_{a}\cdot n_{b}\right)\;I-n_{b}⊗n_{a}\right]\nabla \phi_{b}\;,\\ \partial_{\phi_{a}}\psi_{\mathrm{gd}}\; & = & 0\;,\\ \partial_{\nabla \phi_{a}}\psi_{\mathrm{gd}}\; & = & 2\;\varphi_{\mathrm{gd}}\;n_{a}\times \left(G_{D}^{T}\gamma_{a}\right)=2\;\varphi_{\mathrm{gd}}\;\sum_{b=1}^{g}\left(\gamma_{a}\cdot \gamma_{b}\right)\left[\left(n_{a}\cdot n_{b}\right)\;I-n_{b}⊗n_{a}\right]\nabla \phi_{b}\;, \end{array}$$

are employed in the sequel.

### 2.4. Chemical Energy

For simplicity, attention is restricted here to disordered phases, crystalline regular solid solution theory, and pairwise interaction. In this case,

(19) $$\begin{array}{ccc} f_{\mathrm{hc}}\left(c\right)\; & = & e_{\mathrm{AA}}+\left(e_{\mathrm{BB}}-e_{\mathrm{AA}}\right)\;c+w_{\mathrm{AB}}\;c\left(1-c\right)+R\theta \;\left[\left(1-c\right)\ln\left(1-c\right)+c\ln c\right]\;,\\ f_{\mathrm{gc}}\left(\nabla c\right)\; & = & \nabla c\cdot N_{\mathrm{gc}}\;\nabla c\;, \end{array}$$

hold for the homogeneous $f_{\mathrm{hc}}$ part of the chemical energy per unit mole and its gradient part $f_{\mathrm{gc}}$, respectively. These depend on the molar ab bonding energy $e_{ab}$ (units J mol${}^{-1}$), the relative interaction energy $w_{\mathrm{AB}}:=2e_{\mathrm{AB}}-\left(e_{\mathrm{AA}}+e_{\mathrm{BB}}\right)$, and the energy modulus $N_{\mathrm{gc}}$ (units J m${}^{2}$ mol${}^{-1}$). Note that *R* in (19)${}_{1}$ is the universal gas constant.

### 2.5. Driving Forces for Solute Flux, Chemomechanical Binodal and Spinodal

The current energy model (5)–(7) determines the forms

(20) $$\begin{array}{ccc} \mu \; & = & \partial_{c}f_{\mathrm{el}}+\partial_{c}f_{\mathrm{hd}}+\partial_{c}f_{\mathrm{hc}}-2N_{\mathrm{gc}}\cdot \nabla c\;,\\ \nabla \mu \; & = & {\left(\nabla F\right)}^{T}\;\partial_{c}\partial_{F}f_{\mathrm{el}}+\left(\partial_{c}^{2}f\right)\;\nabla c+{\left(\nabla \phi \right)}^{T}\;\left[\partial_{c}\partial_{\phi }f_{\mathrm{el}}+\partial_{c}\partial_{\phi }f_{\mathrm{hd}}\right]-2\;\nabla \left(N_{\mathrm{gc}}\cdot \nabla c\right)\;, \end{array}$$

for the (chemomechanical) chemical potential μ from the first relation in (4) and its spatial gradient, respectively. In particular, the latter represents thermodynamic force driving solute flux j via the third relation in (4), and so solute segregation. For example, the *c* dependence of $f_{\mathrm{hd}}$ drives Suzuki [28] segregation (i.e., to stacking faults) in the fcc case. Analogously, that of $f_{\mathrm{el}}$ due to solute misfit lies behind Cottrell segregation.

A second consequence of (5)–(7) are the relations

(21) $$\begin{array}{ccccccc} \partial_{c}f\left(c,F,\phi \right)=0\;, & \; & \partial_{c}f\; & = & \partial_{c}f_{\mathrm{ho}}\; & = & \partial_{c}f_{\mathrm{el}}+\partial_{c}f_{\mathrm{hd}}+\partial_{c}f_{\mathrm{hc}}\;,\\ \partial_{c}^{2}f\left(c,F,\phi \right)=0\;, & \; & \partial_{c}^{2}f\; & = & \partial_{c}^{2}f_{\mathrm{ho}}\; & = & \partial_{c}^{2}f_{\mathrm{el}}+\partial_{c}^{2}f_{\mathrm{hd}}+\partial_{c}^{2}f_{\mathrm{hc}}\;, \end{array}$$

(recall that μ=0 implies $\mu_{A}=\mu_{B}$) for the chemomechanical binodal and spinodal hypersurfaces, respectively, in (c,F,ϕ) space. In the current model, then, both the binodal and spinodal deviate from their purely chemical counterparts $\partial_{c}f_{\mathrm{hc}}=0$ and $\partial_{c}^{2}f_{\mathrm{hc}}=0$ due to the solute concentration dependence of the dislocation $f_{\mathrm{hd}}$ and elastic $f_{\mathrm{el}}$ contributions to the energy of the binary alloy. Recall that the former is related to the stacking fault energy in the fcc case, or to the (screw) core spreading energy in the bcc case. In the binodal case, both the non-linear elastic relation in (13) for $\partial_{c}f_{\mathrm{el}}$, and linear elastic relation in (16) for $\partial_{c}f_{\mathrm{le}}$, predict a dependence of the chemomechanical binodal on the (non-linear, linear) lattice strain state via the stress. As shown by the third relation in (13) for $\partial_{c}^{2}f_{\mathrm{el}}$ and the third relation in (16) for $\partial_{c}^{2}f_{\mathrm{le}}$, such a dependence is also predicted for the chemomechanical spinodal by non-linear elastic model, but not by the linear elastic one. These aspects of the current model are examined more closely in the following with the help of simulation.

## 3. Simplified Model for Cubic Crystals

### 3.1. Reduction to Cubic Symmetry

All analytical and simulation results to be discussed in what follows are for the case of cubic single crystals. In this case, the misfit distortion per unit solute concentration $N_{S}$ takes the cubic form

(22) $$N_{S}=\upsilon_{S}I\;,$$

where $\upsilon_{S}$ is the scalar dilatation per unit concentration. Given (22), the non-linear elastic relations in (13) reduce to

(23) $$\begin{array}{ccccc} \partial_{c}\psi_{\mathrm{el}}\; & = & -\upsilon_{S}\;I\cdot K\; & = & -k_{\mathrm{el}}\upsilon_{S}\;\left[\frac{1}{3}I\cdot E_{L}+4\;\psi_{\mathrm{el}}/k_{\mathrm{el}}\right]\;,\\ \partial_{c}^{2}\psi_{\mathrm{el}}\; & = & -\upsilon_{S}\;I\cdot \partial_{c}K\; & = & k_{\mathrm{el}}\upsilon_{S}^{2}\;\left[1+2\;I\cdot E_{L}+16\;\psi_{\mathrm{el}}/k_{\mathrm{el}}\right]\;, \end{array}$$

with $I\cdot A=A_{11}+A_{22}+A_{33}$ and $k_{\mathrm{el}}:=I\cdot C_{\mathrm{el}}I=3\left(C_{11}+2C_{12}\right)$ for cubic symmetry. Analogously, the linear elastic relations (16)${}_{1,3}$ reduce to

(24) $$\partial_{c}\psi_{\mathrm{le}}=-\upsilon_{S}\;I\cdot T=-\frac{1}{3}\;k_{\mathrm{el}}\upsilon_{S}\;I\cdot E_{\mathrm{Ll}}\;,\;\partial_{c}^{2}\psi_{\mathrm{le}}=k_{\mathrm{el}}\upsilon_{S}^{2}\;,$$

via (16)${}_{4}$ in the cubic case. Since $I\cdot H_{\mathrm{Dl}}=0$ for dislocation glide, note that $I\cdot E_{\mathrm{Ll}}=I\cdot H-3\;\nu_{S}\;c$. Since dislocation glide does contribute to $I\cdot E_{L}$, this is another difference between the non-linear and linear models. Additional simplifications in the cubic case include that

(25) $$N_{\mathrm{gc}}=\kappa_{\mathrm{gc}}\;I\;,\;\kappa_{\mathrm{gc}}=\frac{1}{2}\;a_{0}^{2}\;w_{\mathrm{AB}}\;,$$

for $N_{\mathrm{gc}}$ in (19), where $a_{0}$ is the lattice spacing in the solvent, and $\kappa_{\mathrm{gc}}$ the chemical gradient energy coefficient (units J m${}^{2}$ mol${}^{-1}$). In this case, the chemical energy (19) reduces in essence to the cubic Cahn-Hilliard (CH) form [25]. To emphasize that we are working with the CH model for the chemical energy, let

(26) $$\begin{array}{ccc} f_{\mathrm{CH}}\; & := & f_{\mathrm{hc}}+f_{\mathrm{gc}}\\ \; & = & e_{\mathrm{AA}}+w_{\mathrm{AB}}\;c\left(1-c\right)+R\theta \;\left[\left(1-c\right)\ln\left(1-c\right)+c\ln c\right]+\frac{1}{2}\;a_{0}^{2}\;w_{\mathrm{AB}}\nabla c\cdot \nabla c \end{array}$$

represent the chemical part of *f* in what follows based on $e_{\mathrm{BB}}=e_{\mathrm{AA}}$. Recall that the formulation of Cahn and Hilliard [25] is based on energy per atom, rather than energy per mole as in the current work.

### 3.2. Non-Dimensional Model Relations

Scaling is based as usual in particular on a typical length $\ell_{0}$ (e.g., system size) and time $t_{0}$. In what follows, $g^{*}:=g/g_{0}$ represents the scaled/non-dimensional form of any quantity *g*. In particular, $\nabla^{*}:=\ell_{0}\nabla$ is the non-dimensional gradient operator. Given these, the CH chemical energy (per unit mole) (26) takes the form

(27) $$f_{\mathrm{CH}}=e_{\mathrm{AA}}+w_{\mathrm{AB}}\;\left[c\left(1-c\right)+\frac{1}{2}\;\theta^{*}\;\left(\left(1-c\right)\ln\left(1-c\right)+c\ln c\right)+\frac{1}{2}\;a_{0}^{*}\nabla^{*}c\cdot a_{0}^{*}\nabla^{*}c\right]$$

with $a_{0}^{*}:=a_{0}/\ell_{0}$ and $\theta_{0}:=w_{\mathrm{AB}}/2R$. Likewise, one obtains the scaled form

(28) $${\dot{c}}^{*}=t_{c}^{*\;-1}\;{\mathrm{div}}^{*}\nabla^{*}\mu^{*}\;,\;0={\mathrm{div}}^{*}P^{*}\;,\;{\dot{\phi }}_{a}^{*}=-t_{a}^{*\;-1}\;\delta_{\phi_{a}}\psi^{*}\;,$$

of the model field relations from (1)${}_{1,2}$ and (4)${}_{2,3}$, where

(29) $$t_{c}:=m_{c}^{-1}\ell_{0}^{2}\;\mu_{0}^{-1}\;,\;t_{a}:=m_{a}^{-1}\psi_{0}^{-1}\;,$$

are typical timescales for solute diffusion and dislocation glide, respectively. Note that $m_{c}\ell_{0}^{-1}\mu_{0}$ represents the solute diffusivity corresponding to $m_{c}$.

In the following, the typical length $\ell_{0}$ is determined by the largest system/simulation cell size, e.g., $L_{z}=160\;a_{0}$ in the simulations to be discussed below. For a typical fcc lattice constant $a_{0}=4\times 10^{-10}$ m, for example, this implies $\ell_{0}∼10^{-7}$ m, which is adopted here. In addition, for the case of solute segregation to static dislocations, $m_{a}=0$, $t_{a}=\infty$, and $t_{c}$ is the material timescale of interest. To facilitate investigation of solute interaction with moving dislocations, solute diffusion is assumed to be much faster than dislocation glide, i.e., $t_{c}\ll t_{a}$. In all cases, final results are based on ${\dot{c}}^{*}=0$ and ${\dot{\phi }}_{a}^{*}=0$ on the timescale $t_{0}$.

## 4. Simulation Details

### 4.1. Numerical Solution of Initial-Boundary-Value Problems Based on MPFCM

This is based in particular on the “weak” form Ubachs et al. [29], Shanthraj et al. [30]

(30) $$f_{\mathrm{wgc}}\left(c,\overset{˘}{c},\nabla \overset{˘}{c},\phi \right):=\frac{1}{2}\;\alpha \;{\left(c-\overset{˘}{c}\right)}^{2}+f_{\mathrm{gc}}\left(\nabla \overset{˘}{c}\right)$$

of the gradient chemical energy in terms of the auxiliary field $\overset{˘}{c}$ and penalty parameter α. In this context, the difference between $\overset{˘}{c}$ and *c* is minimized via minimization of the last two terms in (30) with respect to $\overset{˘}{c}$. As usual, the corresponding Euler-Lagrange relation

(31) $$\delta_{\overset{˘}{c}}f_{\mathrm{wgc}}=\alpha \;\left(\overset{˘}{c}-c\right)-2\;\kappa_{\mathrm{gc}}\mathrm{div}\nabla \overset{˘}{c}=0$$

is necessary for this and provides a field relation for $\overset{˘}{c}$. In the context of (30) and (31), note that $\mu =\partial_{c}f_{\mathrm{ho}}+\alpha \;\left(c-\overset{˘}{c}\right)$ approximates the first relation in (20) for the chemical or diffusion potential.

Numerical solution of the independent field relations in (1) and (31) is carried out in a staggered fashion. Initial conditions here include uniform solute concentration in each case. Boundary conditions include zero external loading (stress control). Iteration proceeds until $\dot{c}=0$. To minimize the difference $c-\overset{˘}{c}$, a large value $\alpha^{*}=10^{8}$ of $\alpha^{*}$ is employed in all simulations. Changing this value an order of magnitude either way has no influence on the simulation results.

As in the case of previous applications of MPFCM, e.g., to the modeling of dislocation-solute interaction in Ni-Al-Co in [23], the model is implemented as a module in the simulation software toolkit DAMASK. This is an open-source toolkit for the numerical solution of initial-boundary-value problems based on coupled field relations like (1)${}_{1,2}$ and (4)${}_{4}$ with (4)${}_{1-3}$. Numerical solution based on both finite-element and spectral methods is employed. For more information, the interested reader is referred to the DAMASK website https://damask.mpie.de (accessed on 1 April 2021).

### 4.2. Simulation Set-Up

Unless otherwise stated, all simulation cells are fully periodic and cubic with cell side vectors $\left(L_{x}i_{x},L_{y}i_{y},L_{z}i_{z}\right)$. In the case of fcc edge dislocations, for example,

(32) $$\left(i_{x},i_{y},i_{z}\right)=\left(\frac{1}{\sqrt{2}}\left[\bar{1}10\right],\frac{1}{\sqrt{3}}\left[111\right],\frac{1}{\sqrt{6}}\left[11\bar{2}\right]\right).$$

Dislocation simulations assume initially perfect edge dislocation dipoles with glide plane normal $i_{y}$.

## 5. Results

### 5.1. Linear Chemoelastic Binodal and Spinodal in Defect-Free Cubic Crystals

In this case, the dislocation contributions $\psi_{\mathrm{hd}}$ and $\psi_{\mathrm{gd}}$ to ψ are zero, and (5)–(7) reduce to $\psi =\psi_{\mathrm{el}}+\psi_{\mathrm{hc}}+\psi_{\mathrm{gc}}$, with the sum of the latter two given by (26) for $f_{\mathrm{CH}}$. Note that the homogeneous chemical part of (27) for $f_{\mathrm{CH}}^{*}$ determines the forms

(33) $$\theta_{\mathrm{CHb}}^{*}\left(c\right)=\frac{\left(1-2c\right)}{\tanh^{-1}\left(1-2c\right)}\;,\;\theta_{\mathrm{CHs}}^{*}\left(c\right):=4\;c\left(1-c\right)\;,\;c_{\mathrm{CHs}\pm }\left(\theta^{*}\right)=\frac{1}{2}\pm \frac{1}{2}\sqrt{1-\theta^{*}}\;,$$

for the non-dimensional chemical binodal temperature $\theta_{\mathrm{CHb}}^{*}$, the non-dimensional chemical spinodal temperature $\theta_{\mathrm{CHs}}^{*}$, and the chemical spinodal points $c_{\mathrm{CHs}\pm }$, respectively. In the linear chemoelastic case and (24), note that (33) generalize to the chemoelastic forms

(34) $$\begin{array}{ccc} \theta_{\mathrm{leb}}^{*}\left(d\left(H\right),c\right)\; & = & \left(1-\frac{1}{2}\frac{v_{m}k_{\mathrm{el}}\nu_{S}}{w_{\mathrm{AB}}}\nu_{S}\right)\theta_{\mathrm{CHb}}^{*}\left(c\right)+\frac{1}{2}\frac{v_{m}k_{\mathrm{el}}\nu_{S}}{w_{\mathrm{AB}}}\;\frac{\nu_{S}-\frac{2}{3}\;d\left(H\right)}{\tanh^{-1}\left(1-2\;c\right)}\;,\\ \theta_{\mathrm{les}}^{*}\left(c\right)\; & = & \theta_{\mathrm{CHs}}^{*}\left(c\right)-\frac{1}{2}\frac{v_{m}k_{\mathrm{el}}\nu_{S}}{w_{\mathrm{AB}}}\nu_{S}\;4\;c\left(1-c\right)\;,\\ c_{\mathrm{les}\pm }\left(\theta^{*}\right)\; & = & \frac{1}{2}\pm \frac{1}{2}{\left[1-\theta^{*}/\left(1-\frac{1}{2}\frac{v_{m}k_{\mathrm{el}}\nu_{S}}{w_{\mathrm{AB}}}\nu_{S}\right)\right]}^{\frac{1}{2}}\;, \end{array}$$

for dilatation d(H):=I·H control, and those

(35) $$\begin{array}{ccc} \theta_{\mathrm{leb}}^{*}\left(\sigma_{h}\left(T\right),c\right)\; & = & \theta_{\mathrm{CHb}}^{*}\left(c\right)-3\;\frac{v_{m}k_{\mathrm{el}}\nu_{S}}{w_{\mathrm{AB}}}\;\frac{\sigma_{h}^{*}\left(T\right)}{\tanh^{-1}\left(1-2\;c\right)}\;,\\ \theta_{\mathrm{les}}^{*}\left(c\right)\; & = & \theta_{\mathrm{CHs}}^{*}\left(c\right)\;,\\ c_{\mathrm{les}\pm }\left(c\right)\; & = & c_{\mathrm{CHs}\pm }\left(c\right)\;, \end{array}$$

for (non-dimensional) hydrostatic stress $\sigma_{h}^{*}\left(T\right):=\frac{1}{3}\;k_{\mathrm{el}}^{-1}I\cdot T$ control. Clearly, (34) and (35) reduce to (33) for $\upsilon_{S}\to 0$. Recall that (34), and in particular (35), are based on neglecting the dependence of $C_{\mathrm{el}}$ and $\upsilon_{S}$ on *c*.

**Figure 1:**
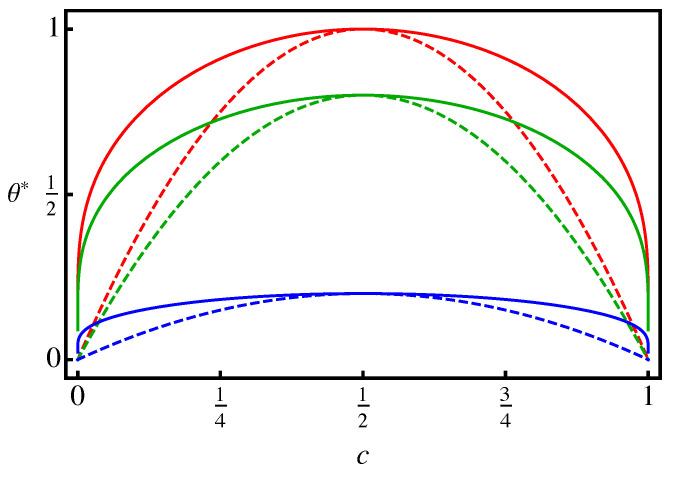
Linear chemoelastic binodal $\theta_{\mathrm{leb}}^{*}\left(c,\frac{3}{2}\upsilon_{S}\right)$ (solid curves) and spinodal $\theta_{\mathrm{les}}^{*}\left(c\right)$ (dashed curves) for a solute misfit of $\upsilon_{S}=0$ (red), $\upsilon_{S}=0.02$ (green), $\upsilon_{S}=0.04$ (blue), with $v_{m}\;k_{\mathrm{el}}/w_{\mathrm{AB}}=10^{3}$.

**Figure 2:**
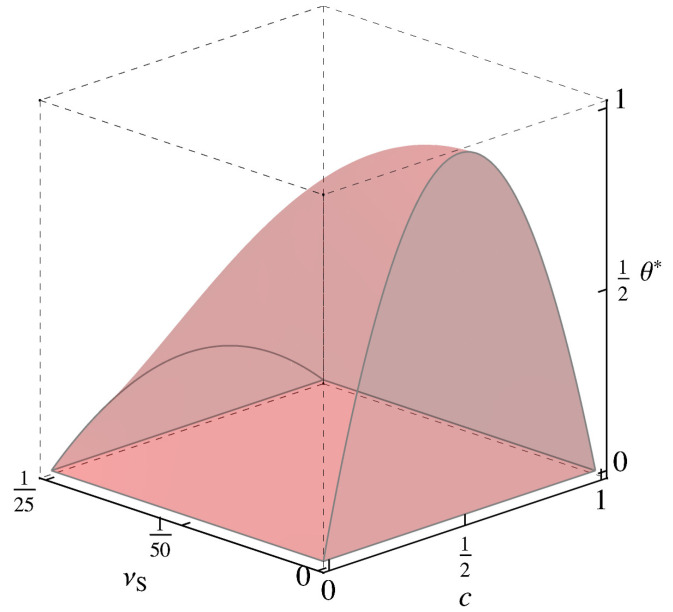
Linear chemoelastic ($f=f_{\mathrm{le}}+f_{\mathrm{CH}}$) spinodal region $\partial_{c}^{2}f<0$ and surface $\partial_{c}^{2}f=0$ in composition *c*, solute misfit $\upsilon_{S}$, normalized temperature $\theta^{*}$, space for $v_{m}\;k_{\mathrm{el}}/w_{\mathrm{AB}}=10^{3}$.

Recall that $\lim_{\;c\to \frac{1}{2}}\theta_{\mathrm{CHb}}^{*}\left(c\right)=1=\lim_{\;c\to \frac{1}{2}}\theta_{\mathrm{CHs}}^{*}\left(c\right)$ represents the so-called (here lower) critical (solid) solution or consolute temperature. In the linear chemoelastic case, $\lim_{\;c\to \frac{1}{2}}\theta_{\mathrm{leb}}^{*}\left(d\left(H\right),c\right)$ diverges for $\upsilon_{S}\neq 0$ except at the “critical” dilatation $d\left(H\right)=\frac{3}{2}\;\upsilon_{S}$. Indeed, at this dilatation, the second term in (34)${}_{1}$ vanishes, and $\lim_{\;c\to \frac{1}{2}}\theta_{\mathrm{leb}}^{*}\left(c,\frac{3}{2}\upsilon_{S}\right)=1-\frac{1}{2}\;v_{m}\;k_{\mathrm{el}}\nu_{S}^{2}/w_{\mathrm{AB}}$ holds. Note that this dilatation corresponds to $I\cdot T=\frac{1}{3}\;k_{\mathrm{el}}\;I\cdot E_{\mathrm{Ll}}=\frac{1}{2}\;k_{\mathrm{el}}\upsilon_{S}\;\left(1-2c\right)$. In this case, $\lim_{\;c\to \frac{1}{2}}\sigma_{h}^{*}\left(T\right)/\tanh^{-1}\left(1-2\;c\right)=\frac{1}{6}\;\nu_{S}$; (34)${}_{1}$ and (35)${}_{1}$ are then consistent. Figure 1 displays $\theta_{\mathrm{leb}}^{*}\left(c,\frac{3}{2}\upsilon_{S}\right)$ and $\theta_{\mathrm{les}}^{*}\left(c\right)$ for selected values of $\upsilon_{S}$. At least at the critical dilatation, then, the binodal region (miscibilty gap) and its spinodal counterpart decrease with increasing solute misfit. For the spinodal region, this is also shown in Figure 2. Clearly, there is a signficant effect of elasticity, and in particular of solute misfit, on the binodal and spinodal in the context of linear chemoelasticity.

In the non-linear chemoelastic case $\psi =\psi_{\mathrm{el}}+\psi_{\mathrm{CH}}$, the corresponding binodal and spinodal are determined by (23)${}_{1}$ for $\partial_{c}\psi_{\mathrm{el}}$ rather than by $\partial_{c}\psi_{\mathrm{le}}$ from (24)${}_{1}$. This non-linear form is employed in all simulations in the sequel. Further, these are based on the scaling choices

(36) $$\mu_{0}=\upsilon_{m}\;k_{\mathrm{el}}\nu_{S}\;,\;\psi_{0}=k_{\mathrm{el}}\;,$$

for the driving forces in (28)${}_{1,3}$. The value $w_{\mathrm{AB}}^{*}:=w_{\mathrm{AB}}/v_{m}\;k_{\mathrm{el}}=10^{-3}$ of $w_{\mathrm{AB}}^{*}$ employed in Figure 1 and Figure 2 is adopted as well in what follows. Further, we work with $\theta^{*}=0.5$, and the typical values $C_{11}/\psi_{0}=1.5\times 10^{-1}$, $C_{12}/\psi_{0}=9\times 10^{-2}$, $C_{44}/\psi_{0}=8\times 10^{-2}$, and $\nu_{S}=2\times 10^{-2}$, for an fcc metal. In this case, note that $\psi_{\mathrm{el}}^{*}∼10^{-4}$ for $|E_{L}|∼10^{-2}$. These and other typical non-dimensional values employed in the simulations to follow are listed in Table 1.

The last three non-dimensional parameter values are related to dislocation dissociation (i.e., $\psi_{\mathrm{hd}\;0}^{*}$) and core energy (i.e., $\psi_{\mathrm{gd}\;0}^{*}$, $\psi_{\mathrm{gd}\;1}^{*}$) relevant to the case of solute interaction with a gliding dislocation and discussed in more detail in Section 5.4 below. Note that the enhanced solute mobility at the dislocation core is neglected in this work, i.e., solute mobility $m_{c}$ does not depend on dislocation order parameters ϕ. However, as it will be shown in the dynamic case, solute drag due to dislocation motion is automatically captured by the model.

### 5.2. Single Static Perfect Edge Dislocation

For simplicity, the simulation examples to be discussed in the following three subsections neglect the dislocation core energy $\psi_{\mathrm{gd}}$ in (7). In addition, dislocations involved are assumed to be of perfect Peierls-Nabarro (PN)-type, in which case $\psi_{\mathrm{hd}}$ is of Frenkel potential-type (e.g., Hirth and Lothe [2], Schoeck [31]). Then g=1, and the planar dislocation phase field/disregistry $\phi_{1}\left(x\right)=\phi_{\mathrm{PN}}\left(\gamma_{1}\cdot x\right)$ is determined by the analytic PN arctan-based disregistry $\phi_{\mathrm{PN}}$ (e.g., Hirth and Lothe [2]). Note for example that $\gamma_{1}=\sqrt{3/2}\;s_{1}$ in the fcc case. Further, $m_{a}=0$ ($t_{a}=\infty$) in the static case as mentioned above. Lastly, an initially uniform solute concentration with c(0)=0.11 is assumed in all cases.

**Figure 3:**
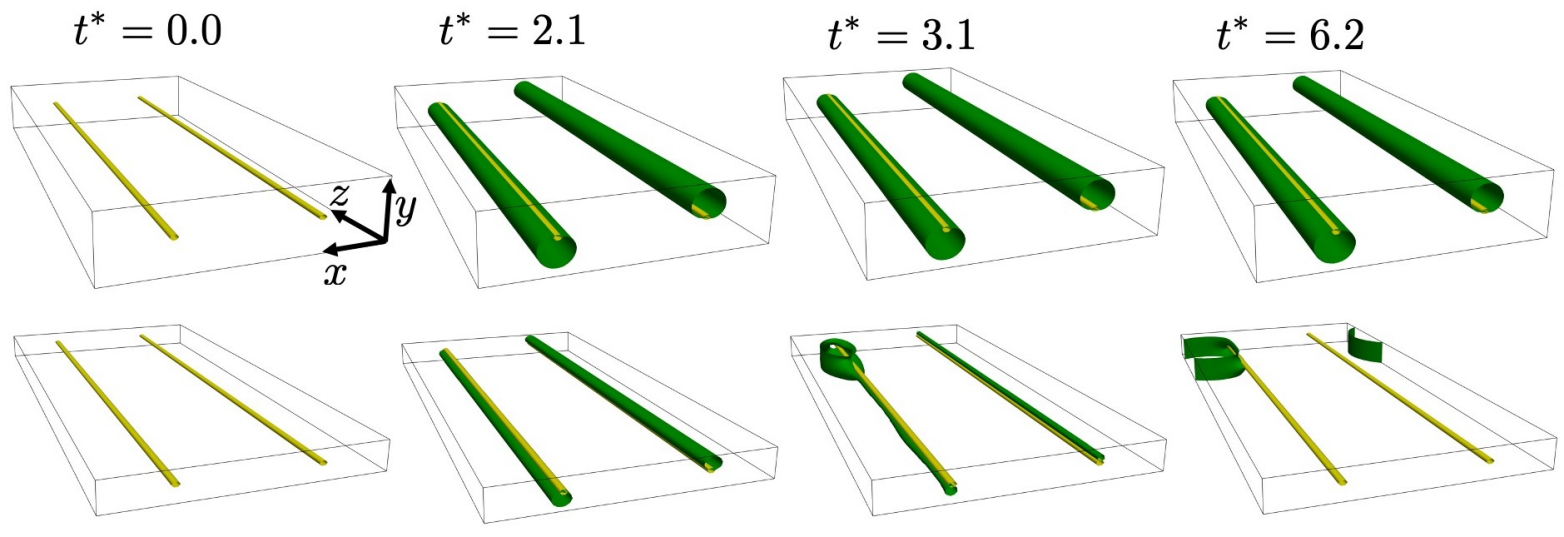
Snapshots of solute segregation to a perfect Peierls-Nabarro edge dislocation dipole for larger $\left(L_{x},L_{y},L_{z}\right)=\left(80,20,160\right)\;a_{0}$ (above) and smaller $\left(L_{x},L_{y},L_{z}\right)=\left(80,10,160\right)\;a_{0}$ (below) simulation cells. The cell orientation is as given in (32). Dislocation lines are shown in yellow, and the 40% solute concentration isosurface in green, and $t_{0}=10^{2}t_{c}$. Note that segregated solute is below (above) the left (right) monopole, corresponding to the region of positive hydrostatic stress. See text for discussion.

Results for segregation to a perfect PN edge dislocation for two system/simulation cell sizes are shown in Figure 3.

Initially, solute segregation to the dipoles results in a uniform solute distribution (with maximum concentration c=0.97) along the lines in both systems (left two snapshots). Whereas this distribution is stable in the larger system (above) up to $t^{*}=6.2$, it decomposes into a single cylindrical precipitate at one of the monopoles in the smaller one (below). In contrast to the larger system (above), the smaller system (below) contains too little solute (about $5.4\times 10^{5}$ solute atoms below, $1.1\times 10^{6}$ atoms above) for segregation alone to stabilize the uniform solute distribution along the monopoles against spinodal decomposition and precipitation for $t^{*}>2.1$. From the point of view of statistical thermodynamics, the larger system is more grand-canonical-like, and the smaller more canonical-like, in its behavior.

### 5.3. Low-Angle Grain Boundary

#### 5.3.1. Tilt Boundary

**Figure 4:**
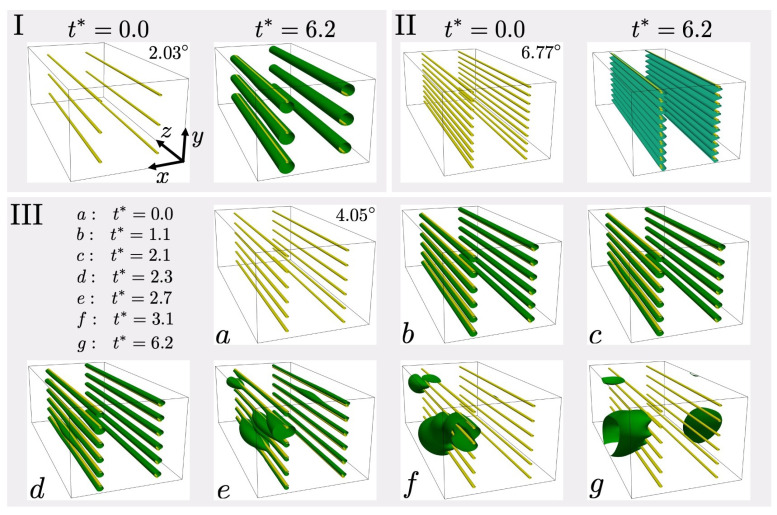
Snapshots of solute segregation to three different low-angle tilt grain boundaries. As before, dislocation lines are displayed in yellow, and solute 40% concentration iso-surfaces in dark green. In (II), the 10% solute iso-surface is displayed in light green, and $t_{0}=10^{2}t_{c}$.

Consider next the case of solute segregation to, and precipitation at, an array of static PN edge dislocation dipoles of the type from the last subsection. To this end, a simulation cell of size $\left(L_{x},L_{y},L_{z}\right)=\left(80,60,160\right)a_{0}$ is employed. Three glide plane/dipole spacings of $d=6\;a_{0}$, $d=10\;a_{0}$ and $d=20\;a_{0}$ result in low-angle grain boundaries (LAGBs) of tilt angle $6.77^{\circ }$, $4.05^{{}^{\circ}}$, and $2.03^{\circ }$, respectively. Results for segregation of an initially uniform solute distribution to these arrays are displayed in Figure 4.

For case (I) with tilt angle $2.03^{{}^{\circ}}$, the separation between the dipoles is such that their interaction is relatively low, and segregation takes place to each as an essentially isolated dipole. In addition, the system size is sufficiently large for the uniform segregated solute distributioin to stabilize against spinodal decomposition and preciptate formation along the line, analogous to the behavior in Figure 3 (top row). For case (II) with tilt angle $6.77^{{}^{\circ}}$, the dipoles are sufficiently close to each other that their stress fields shield each other, leading to an effective reduction of stress field strength and less segregation. Indeed, the maximum solute concentration here is about 0.37, which is much lower than in cases (I) and (III).

For the intermediate case (III) of tilt angle $4.05^{{}^{\circ}}$, the situation is similar to that of the single static dislocation in Figure 3 (bottom row). Indeed, increasing the number of dislocations in the system at constant system size effectively reduces the system size per dislocation. Note that the dislocation density is $7.8\times 10^{15}$ in (I), $2.6\times 10^{16}$ in (II), and $1.6\times 10^{16}$ 1/m${}^{2}$ in (III). Consequently, solute content limitation is again stronger, and segregation alone cannot stabilize the initially uniform solute distribution along the monopoles against spinodal decomposition. As seen starting at $t^{*}=2.3$ in Figure 4e–g, because of this, the uniform distribution along the lines becomes unstable and precipitate formation leads to solute depletion along the lines. The resulting precipitates have maximum solute concentration close to the bulk binodal (about 0.9).

#### 5.3.2. Twist Boundary

**Figure 5:**
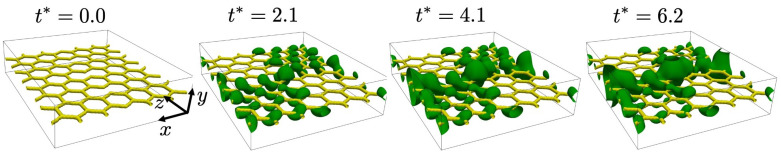
Solute segregation to low angle twist grain boundary as a hexagonal network of screw dislocations. Yellow indicated the dislocation lines, dark green is solute iso-surface of 0.4, and $t_{0}=10^{2}t_{c}$.

Analogous simulation is carried out for the case of low angle twist boundary. These boundaries often result in a network of screw dislocations, in this case a hexagonal network. Note that to satisfy the periodicity, the simulation box is divided into four sections with four twist boundaries with opposite twist angles, resulting in zero sum (analogous to the tilt boundary dipole above). The snapshots of this simulation are shown in Figure 5.

In contrast to (linear elastic) continuum dislocation theory, non-linear effects accounted for in MPFCM result in a non-zero hydrostatic stress field in screw cores driving segregation to these as well. Note that atomistic modeling based on hybrid Monte Carlo molecular dynamics (Sadigh et al. [16]), or on diffusive molecular dynamics (e.g., Dontsova et al. [32], Ponga and Sun [33]), also predict segregation to screw dislocations. Again, this is in contrast to continuum modeling based on linear elasticity.

Although much smaller than its edge counterpart, the hydrostatic screw stress field is sufficient to drive Cottrell segregation to these as well. As shown in Figure 5, this is also the case for screw configurations like a twist boundary. In this latter case, maximum positive hydrostatic stress, and so segregation, appears to be at junctions where four sections meet and the twist angle is reversed. Due to the completely different stress field of the screw configuration in the twist boundary, note that the segregated solute distribution is apparently immediately unstable and decomposes into precipitates from the start, in contrast to the single static edge dislocation (Figure 3) and tilt boundary (Figure 4) cases.

### 5.4. Single Gliding Dislocation

As a last application, consider solute segregation to, and interaction with, a single gliding dislocation. In contrast to the previous examples, the dislocation energies $\psi_{\mathrm{hd}}$ in (6) and $\psi_{\mathrm{gd}}$ in (18) now play a role. Focusing attention here on the fcc case, for a single edge dislocation, g=2, $\left(s_{1},n_{1,2},s_{2}\right)=\left(i_{x},i_{y},i_{z}\right)$ from (32), $b_{1}=a_{0}/\sqrt{2}$, $b_{2}=a_{0}/\sqrt{6}$, $d:=d_{1,2}=a_{0}/\sqrt{3}$ and $\phi =(\phi_{1},\phi_{2})$. Then $\psi_{\mathrm{hd}}\left(c,\phi \right)$ reduces to $\psi_{\mathrm{hd}}\left(c,\phi_{1},\phi_{2}\right)=\psi_{\mathrm{hd}0}\;\gamma_{\mathrm{sf}}^{*}\left(c,\phi_{1},\phi_{2}\right)/d^{*}$, and $\psi_{\mathrm{gd}}\left(\nabla \phi \right)$ to $\psi_{\mathrm{gd}}\left(\nabla \phi_{1},\nabla \phi_{2}\right)=\sum_{a=1}^{2}\psi_{\mathrm{gd}\;a}\;\nabla^{*}\phi_{a}\cdot \left(I-n_{a}⊗n_{a}\right)\nabla^{*}\phi_{a}$ with $\psi_{\mathrm{gd}\;a}:=\varphi_{\mathrm{gd}}\ell_{0}^{-2}\;\gamma_{a}^{2}$. In the first of these, $\gamma_{\mathrm{sf}}\left(c,\phi_{1},\phi_{2}\right)$ is the fcc stacking fault energy whose representation is given for example in Mianroodi et al. [23]. For simplicity, the *c* dependence of $\gamma_{\mathrm{sf}}^{*}\left(c,\phi_{1},\phi_{2}\right)/d^{*}$ is assumed linear with scaled slope of 3 times the value of $\psi_{\mathrm{hd}\;0}^{*}$ in Table 1, resulting in a negative driving force for Suzuki segregation to stacking faults.

**Figure 6:**
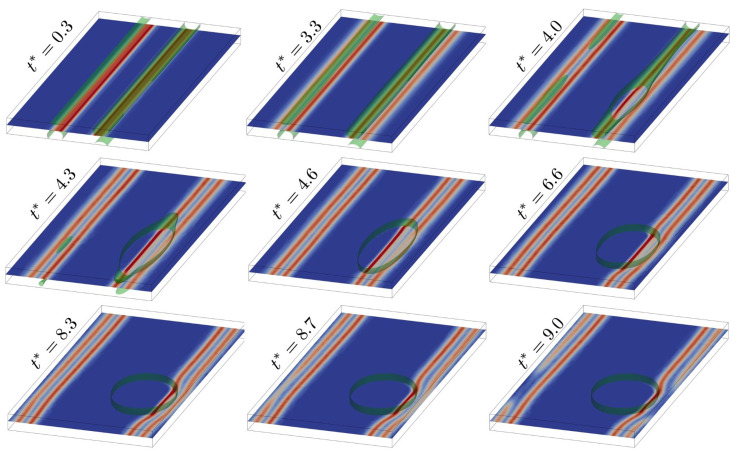
Solute segregation to, and spinodal decomposition at, an fcc edge dislocation gliding under an external shear deformation ${\bar{F}}_{xy}=0.10$ in a system/simulation cell with orientation (32). 40% solute concentration isosurface shown in green. Dislocation line visualization is based on the scalar field $|\nabla \phi_{1}|+|\nabla \phi_{2}|$, which varies between zero (blue) and maximal (red) in the dislocation core. Note that the external shear deformation is applied at a rate much faster than $t_{c}^{-1}$. In addition, $t_{c}=10^{-2}t_{a}$, and $t_{0}=10^{2}t_{a}$. See text for discussion.

The corresponding results are shown in Figure 6.

At the start ($t^{*}=0.3$), solute segregates to the initially perfect dislocation dipole. Each monopole of the dipole then dissociates into Shockley partials ($t^{*}=3.3$) and begins to glide ($t^{*}=4.0$). In the process, the initially uniform solute distribution along each monopole destabilizes, driven in part by negative Suzuki segregation. Due to the boundary conditions, this takes the form of complete solute depletion along the left dissociated monopole, precipitate formation at the right dissociated monopole, and solute depletion along the rest of this monopole ($4.0⩽t^{*}⩽6.6$). The higher stacking fault energy inside the precipitate results in a reduction of the stacking fault width between the partial dislocations in the right monopole and a bend in the dislocation lines at the precipitate interface.

Under the current boundary conditions, the force due to spinodal instability driving precipitate formation is stronger that the Cottrell force on solutes due to misfit and the positive hydrostatic core stress field attracting them to the dislocation line. Note that solute distribution due to precipitation increases the spatial separation between solute in the precipitate and the dislocation core, resulting in a reduction of the Cottrell force on solute and no solute transport due to dislocation glide ($t^{*}⩾4.6$). Lastly, as glide continues ($t^{*}>8.3$), the leading partials on both sides interact with their periodic images, resulting in partial annihilation of the leading partials ($t^{*}=9.0$).

## 6. Summary and Discussion

Microscopic phase-field chemomechanics (MPFCM) has been employed in the current work to model solute segregation, dislocation-solute interaction, spinodal decomposition, and precipitate formation, at straight dislocations and configurations of these in a model binary solid alloy. In particular, (i) a single static edge dipole, (ii) arrays of static dipoles forming low-angle tilt (edge) and twist (screw) grain boundaries, as well as at (iii) a moving (gliding) edge dipole, have been considered. MPFCM is formulated for such an alloy in the first part of the work. Central here is the MPFCM model for the alloy free energy, which includes solute, dislocation, and elastic lattice, contributions. Due to solute lattice misfit, the latter energy is concentration dependent, resulting in a strong elastic influence on the binodal and spinodal behavior of the alloy. In addition, MPFCM-based modeling of energy storage couples the thermodynamic forces driving (Cottrell and Suzuki) solute segregation, precipitate formation and dislocation glide. As implied by the simulation results for edge dislocation dipoles and their configurations, there is a competition between (i) Cottrell segregation to dislocations resulting in a uniform solute distribution along the line, and (ii) destabilization of this distribution due to low-dimensional spinodal decomposition when the segregated solute content at the line exceeds the spinodal value locally, i.e., at and along the dislocation line. Due to the completely different stress field of the screw dislocation configuration in the low-angle twist boundary, the segregated solute distribution is immediately unstable and decomposes into precipitates from the start.

Like in previous works based on linear elasticity, the dependence of the elastic energy on solute misfit in the current non-linear treatment is central to the influence of elasticity on (Cottrell) segregation, spinodal decomposition, and precipitate formation. As shown by the treatment in Section 5.1, in this case, the binodal and spinodal depend in a constitutive fashion on the strain or stress (i.e., in addition to the solute concentration) in the (linear) chemoelastic context. In particular, this dependence holds in the case of spatially homogeneous solute concentration, strain and stress, the latter satisfying mechanical equilibrium trivially. On the other hand, again in the chemoelastic context, spinodal decomposition represents a transition from spatially homogeneous to inhomogeneous solute concentration, strain and stress. This was realized by Cahn [10] in his ground-breaking work on the role of solute misfit in the spinodal behavior of defect-free metallic alloys. Under the assumption that spinodal decomposition takes place in mechanical equilibrium, he showed that this process is affected by dependence of the (equilibrium) elastic energy on solute misfit not accounted for in the CH model [25].

To discuss this in more detail, consider the split $\phi =\bar{\phi }+\tilde{\phi }$ of any field ϕ into mean $\bar{\phi }:=\langle \phi \rangle$ (i.e., volume averaged, spatially constant) and spatially fluctuating $\tilde{\phi }$ parts. In this context, Cahn [10] assumed (i) spatial inhomogeneity in one dimension (*x*), (ii) isotropic linear elasticity, (iii) no defects, (iv) $E_{\mathrm{Sl}}=\upsilon_{S}\;\tilde{c}\;I$, (v) $E=E_{xx}\;i_{x}⊗i_{x}$, and (vi) $T=T_{xx}\;i_{x}⊗i_{x}+T_{yy}\;i_{x}⊗i_{y}+T_{zz}\;i_{x}⊗i_{z}$ for the stress. Under these assumptions, mechanical equilibrium reduces to $\mathrm{div}T=T_{xx,x}\;i_{x}=0$. Choosing then $E_{\mathrm{Ll}}=E-E_{\mathrm{Sl}}$ in such a way that $T_{xx}=0$, Cahn [10] obtained ${\bar{\psi }}_{\mathrm{le}}=\upsilon_{S}^{2}\;E\;\langle {\tilde{c}}^{2}\rangle /\left(1-\nu \right)$, with *E* Young’s modulus, and ν Poisson’s ratio. More recently, Onuki [34,35] extended the treatment of Cahn [10] to multiple dimensions and a dependence of the isotropic elastic constants on solute concentration. As discussed for example by Binder and Fratzl [36], the original 1D treatment of the cubic anisotropic case by Cahn [11] has been extended by Khachaturyan [12] and others to 3D and general anisotropy with the help of the Green-function-based formal solution (See also [37]; also used in PFM: e.g., [8,9,38].) of linear elastostatic mechanical equilibrium divT=0. In particular, this yields the form (Here and in what follows, the operator ∗ represents *both* convolution and linear mapping.) $E_{\mathrm{Ll}}={\bar{E}}_{\mathrm{Ll}}+M_{\mathrm{le}}*{\tilde{E}}_{\mathrm{Rl}}$ for the equilibrium lattice strain with $M_{\mathrm{le}}:=\Gamma_{\mathrm{le}}C_{\mathrm{el}}-I$, where $\Gamma_{\mathrm{le}}$ is the linear elastostatic Lippmann-Schwinger operator (${\hat{\Gamma }}_{\;\mathrm{le}}\left(k\right)\;A:=\mathrm{sym}\;\left({\hat{G}}_{\mathrm{le}}\left(k\right)\;A\left(k⊗k\right)\right)$, with $G_{\mathrm{le}}$ the corresponding Green function (${\hat{G}}_{\mathrm{le}}^{-1}\left(k\right)\;a:=C_{\mathrm{el}}\left[a⊗k\right]k$).) (e.g., [39]). In turn, ${\bar{\psi }}_{\mathrm{le}}=\frac{1}{2}\;{\bar{E}}_{\mathrm{Ll}}\cdot C_{\mathrm{el}}{\bar{E}}_{\mathrm{Ll}}+\frac{1}{2}\langle {\tilde{E}}_{\mathrm{Rl}}\cdot A_{\mathrm{le}}*{\tilde{E}}_{\mathrm{Rl}}\rangle$ follows with ${\hat{A}}_{\mathrm{le}}\left(k\right):={\hat{M}}_{\mathrm{le}}^{T}\left(k\right)\;C_{\mathrm{el}}\;{\hat{M}}_{\mathrm{le}}\left(-k\right)$. For the current case and $H_{\mathrm{Rl}}=c\;N_{S}+\sum_{a=1}^{g}\phi_{a}\;N_{Da}$, then, ${\bar{\psi }}_{\mathrm{le}}$ is determined in particular by the term $\frac{1}{2}\langle \tilde{c}\;N_{S}\cdot A_{\mathrm{le}}*\tilde{c}\;N_{S}\rangle$, representing a 3D anisotropic generalization of the above result of Cahn [10]. In addition, one obtains the elastic contribution $\delta_{c}{\bar{\psi }}_{\mathrm{le}}=-N_{S}\cdot \bar{T}-N_{S}\cdot A_{\mathrm{le}}*{\tilde{E}}_{\mathrm{Rl}}$ to the solute chemical/diffusion potential μ satisfying mechanical equilibrium in the spatially inhomogeneous case, in contrast to its constitutive counterpart $\partial_{c}\psi_{\mathrm{le}}=-N_{S}\cdot T$ from (16)${}_{1}$. This is likewise the case for the elastic contribution $\delta_{c}^{2}{\bar{\psi }}_{\mathrm{le}}=N_{S}\cdot C_{\mathrm{el}}N_{S}+N_{S}\cdot A_{\mathrm{le}}*N_{S}$ to the chemoelastic spinodal. This is of course also true for all simulation results in the current work based on elastic non-linearity and MPFCM. Further analogous generalizations of the treatment of “closed” and “open” solid solution chemoelasticity in Larché and Cahn [40] to (i) non-linear chemoelasticity and (ii) non-ideal (e.g., regular) solutions, are also possible and represent work in progress.

## Figures and Tables

**Table 1:** Typical non-dimensional parameter values adopted in the simulations.

$a_{0}^{*}$	$C_{11}^{*}$	$C_{12}^{*}$	$C_{44}^{*}$	$\upsilon_{S}$	$w_{\mathrm{AB}}^{*}$	$\theta^{*}$	$\psi_{\mathrm{hd}\;0}^{*}$	$\psi_{\mathrm{gd}\;1}^{*}$	$\psi_{\mathrm{gd}\;2}^{*}$
$10^{-2}$	$1.5\times 10^{-1}$	$9\times 10^{-2}$	$8\times 10^{-2}$	$2\times 10^{-2}$	$10^{-3}$	0.5	$10^{-4}$	$10^{-6}$	$10^{-7}$

## Data Availability

The data presented in this study are available on request from the corresponding author.

## Abbreviations

The following abbreviations are used in this manuscript:

CH	Cahn-Hilliard
MPFCM	Microscopic phase-field chemomechanics
PFM	Phase-field microelasticity
PN	Peierls-Nabarro
PK	Piola-Kirchhoff
LAGB	low-angle grain boundary
